# The effect of hospital mergers on long-term sickness absence among hospital employees: a fixed effects multivariate regression analysis using panel data

**DOI:** 10.1186/1472-6963-14-50

**Published:** 2014-02-03

**Authors:** Lars Erik Kjekshus, Vilde Hoff Bernstrøm, Espen Dahl, Thomas Lorentzen

**Affiliations:** 1 Department of Health Management and Health Economics, Institute of Health and Society, Faculty of Medicine, University of Oslo, Forskningsveien 3a, 0373 Oslo, Norway; 2 Faculty of Social Science, Oslo and Akershus University College of Applied Sciences, St. Olavs Plass, 0130 Oslo, Norway; 3 Department of Sociology, Faculty of Social Sciences, University of Bergen, Nygårdsgt, 55020 Bergen, Norway

**Keywords:** Merger, Organizational change, Hospital, Sickness absence, Panel data

## Abstract

**Background:**

Hospitals are merging to become more cost-effective. Mergers are often complex and difficult processes with variable outcomes. The aim of this study was to analyze the effect of mergers on long-term sickness absence among hospital employees.

**Methods:**

Long-term sickness absence was analyzed among hospital employees (N = 107 209) in 57 hospitals involved in 23 mergers in Norway between 2000 and 2009. Variation in long-term sickness absence was explained through a fixed effects multivariate regression analysis using panel data with years-since-merger as the independent variable.

**Results:**

We found a significant but modest effect of mergers on long-term sickness absence in the year of the merger, and in years 2, 3 and 4; analyzed by gender there was a significant effect for women, also for these years, but only in year 4 for men. However, men are less represented among the hospital workforce; this could explain the lack of significance.

**Conclusions:**

Mergers has a significant effect on employee health that should be taken into consideration when deciding to merge hospitals. This study illustrates the importance of analyzing the effects of mergers over several years and the need for more detailed analyses of merger processes and of the changes that may occur as a result of such mergers.

## Background

Medical breakthroughs and innovative techniques and equipment have enabled hospitals to treat a greater number of patients. However, these advances have also led to increased costs and the need for more specialized services. Hospitals are striving for growth and higher efficiency. The desire to control rising hospital sector costs, while simultaneously implementing expensive medical advances, has driven managers and politicians to find more efficient ways of organizing hospitals; this has involved hospital mergers. However, the potential impact of such mergers on the workforce has often been left out of planning considerations.

The international hospital sector is experiencing what has been labeled as ‘merger mania’ [1]. The Norwegian hospital sector, in particular, has undergone a high number of hospital mergers. Between 1992 and 2000, more than 27% of Norwegian hospitals were involved in mergers [2]. In 2001, all Norwegian hospitals were transferred from local county to centralized state ownership, which further increased the rate of mergers; from 2000 to 2010, more than 90% of public hospitals were involved in one or more mergers.

The main argument for merging hospitals has been to increase efficiency through economy of scale effects. However, the actual outcome of mergers is disputed [2-4]. A study of Norwegian hospital mergers demonstrated that a single administrative merger can have a significant negative effect on a hospital’s cost-efficiency, whereas more profound mergers that involve a reduction in acute care, may have a positive effect on cost-efficiency [2]. Although merging can benefit efficiency, they may also incur hidden costs. For example, costs arising from increased sickness absence among employees who have been exposed to restructuring processes. A large body of research has documented that workplace reorganization can have adverse effects on employee health [5,6]; the current literature suggests that mergers may also cause similar negative consequences.

Norwegian hospital mergers have not been characterized by major downsizing of personnel. Any potential negative health effects following such organizational changes are unlikely to be caused by job insecurity, but rather by the merger itself and associated change-related activities. The hospital sector accounts for a significant part of the Norwegian labor market, so even small effects on sickness absence rates can have a large impact on resource use across the sector.

### The aim of the study

In the present study, we followed 57 hospitals through more than 20 mergers over a period of 8 years. This study is unique in that we investigated the effect of mergers over a long period, with comparisons across multiple hospitals. The aim of this study was to analyze the effect of mergers on long-term sickness absence among hospital employees. Long-term sickness absence is defined as an absence longer than 16 days due to mental and/or physical ill health. An absence of this duration requires medical certification from a physician; certification is generally considered a valid measure of poor health [7-9]. The analyses were conducted separately for men and women because absence levels tend to be significant higher among female employees [10,11], and prior studies have indicated that females react differently to work environment stressors when compared with men [12].

### Mergers and long-term sickness absence

Several reasons have been proposed for why organizational changes, like mergers, often lead to adverse health effects and increased sickness absence. During large organizational changes, employees often experience added demands because of these changes [13,14]. At the same time, changes may cause employees to feel more uncertain about their jobs and what is expected of them, and how the changes will affect their employment [13,15-17]. The uncertainty and involuntary nature of many change processes often results in employees feeling a loss of control [18,19]. In this way, organizational change can lead to working conditions that are described in the literature as being strained, and possibly harmful to employee health [20,21]. Furthermore, increased levels of conflict have also been postulated as being a potential stressor during change [13], and cultural clashes during mergers are receiving increased attention [22-25]. Mirvis [26] described experiences related to the acquisition of a manufacturing company; this case study illustrated how frustration and conflict can be fueled by differences between the acquiring and acquired firm, i.e. because of different business systems, ways of running the company, and strategies. Vaananen, Pahkin, Kalimo and Buunk [27] demonstrated that how mergers impact an individual employee’s job can affect their post-merger subjective health.

Current literature supports possible negative consequences from mergers and acquisitions, although not conclusively. The potential health effects of mergers have received less attention in the literature than other major organizational changes, such as downsizing.

Probst [28] focused on the merger between five state government agencies involved in providing public health and human services. This merger spawned flourishing rumors of layoffs, although only minor layoffs actually occurred. Employees, identified by management as being affected by the merger, reported poorer physical and mental health 6 months after the merger was announced compared with immediately prior to the announcement. Similarly, Schweiger and Denisi [19] studied the effect of a merger between two plants engaged in light manufacturing. They found that immediately following the merger announcement, the employees’ absence days increased, together with their experience of global stress and uncertainty. Layoffs were a possible consequence of the mergers, because the opportunity to eliminate redundant functions and staff was one of the expected gains from the merger. Brown, Zijlstra and Lyons [29] investigated the effect of restructuring in merged NHS trusts. They found that affected nurses reported significantly higher stress and job pressure compared with non-affected nurses; some affected nurses also reported lower quality of life than non-affected nurses. Lindberg and Rosenqvist [30] focused on two merged Swedish hospitals, which included downsizing and implementation of Total Quality Management (TQM). They found that sick leave in the merged hospital increased more rapidly than in the general Swedish population.

In contrast to the above studies, Westerlund, Ferrie, Hagberg, Jeding, Oxenstierna and Theorell [31] found no significant effect of mergers on long-term sickness absence (absence spells of 90 days or longer) or hospital admissions among 24,036 Swedish employees in public and private organizations. They analyzed the effect of mergers that occurred over a 6-year period (1990–96) on health outcomes during the following 3-year period (1997–99). They found significant negative health effects from large expansions and moderate downsizing, but no effects arising from mergers. The potential lag time between merger exposure and the health measurements (2 months to 9 years) could have led to an underestimation of the effect, if the effect is assumed to be relatively immediate. Because this was a time of downsizing in Sweden, sickness presence could also be a potential confounding variable, indicated by a similar lack of effect of major downsizing on sickness absence rates. Netterstrom, Blond, Nielsen, Rugulies and Eskelinen [32] investigated depression prevalence in Danish municipality and county employees, before and after a reform that merged several municipalities and counties. They found no significant change in depression associated with the merger. Their pre-merger control was measured for 9 months leading up to the merger (and then for another 9 months after the merger announcement). As a consequence, the control could have been affected by the employees’ knowledge of the impending merger.

Previous studies have focused mostly on the effect of one or a few mergers, and sometimes only the first or last phase of the merger. More comprehensive research is needed to investigate the health effects across mergers, and through the whole merger process. The Norwegian hospital sector provides a good case for such research, because of the high frequency of mergers and the availability of reliable longitudinal sickness absence data.

Mergers in the Norwegian health sector are likely to have directly and indirectly affected its employees’ working conditions by increasing the frequency of internal changes. First, they were initiated from above and outside the main organization with the merger forced upon the organization by the regional health authority. The employees’ control over the decision-making process was likely to be low. In this way, the mergers may have directly affected the employees’ working conditions, in particular by increasing their uncertainty and loss of control. Second, the mergers were introduced to enable internal changes and trigger change activities to explore economy of scale effects relating to patient treatment and administration [33]. Merging hospitals have been shown to have higher degrees of internal organizational change following mergers compared to hospitals that have not undergone merging [34]. Other studies have demonstrated that a high frequency of internal changes is likely to adversely affect employee health and sickness absence, by further impairing working conditions [35,36]. Finally, several mergers have resulted in more permanent structural changes, such as an additional management level. Furthermore, several units lost their local leader, the new management team being located away from the hospital with less direct contact with employees. Such centralization processes can also increase sickness absence levels [37].

To measure the full effect of mergers on long-term sickness absence, it must be analyzed over an extended period of time. The immediate effects would likely involve feelings of insecurity elicited by the idea of merging; the subsequent effects would take place when the actual change activities are underway.

## Methods

### Setting

Norwegian hospitals have a long history of mergers. Prior to 1972, Norway experienced a large increase in the number of hospitals, which were built without national planning or an overall strategy. However, the hospital structure was soon recognized as being inefficient because there were too many hospitals serving a relatively small population. Furthermore, increasing hospital specialization also increased their size. Because it was difficult for smaller rural hospitals to attract sufficient numbers of specialized personnel, many hospitals began to collaborate. However, the need for stronger collaboration grew and the number of mergers increased rapidly during the 1990s. Between 1980 and 2000, many hospitals were also closed or transformed into nursing homes. At the same time, if a maternity department had too few deliveries, the obstetricians could not receive sufficient delivery care training to ensure sufficient quality and some units closed [38]. These changes led to protests, and the government was accused of centralization and failing to prioritize rural districts. In 2005, central government promised that all local hospitals would be preserved [39], but hospitals continued to merge, and clinical services and functionalities were divided between the merged units. The large number of mergers in Norway and the lack of obvious cost savings have raised questions about whether these changes are adversely affecting employees unnecessarily. In Norway between 2000 and 2009 happened 23 mergers involving 57 hospitals, which were all included in the present study.

### Study population

All public somatic acute care hospitals in Norway that functioned between 2000 and 2008 were included in the analysis; this produced a panel dataset consisting of 60–70 hospitals units over a period of 8 years. Individual study participants were all hospital employees who had worked at the hospital for the entire year studied. Employees who worked at multiple hospitals during the study period were only included for the time spent at the hospital where they were employed for the longest period. The identification of the study population was made possible by register data collected by Statistics Norway. On behalf of the register owners, Statistics Norway collects and merges population-based public registers and prepares them for research purposes. Statistics Norway’s “Events Database” contains longitudinal information covering the full Norwegian population covering practically all movements between and within the labor market, and welfare and educational systems. A key variable that enabled the merging of individual and hospital-level data, were the business register numbers starting from 2000, which was also supplied by Statistics Norway.

A total of 107,209 employees fitted our selection criteria; see Table 1 for participant characteristics. The group was reasonably heterogeneous (e.g. aged from 16 to 75 years; uneducated to PhDs). However, the vast majority was female (79%).

**Table 1:** Descriptive statistics

	**Female**		**Male**	
	**% of N**	**Mean (SD)**	**% of N**	**Mean (SD)**
**N**	**85 002**		**22 296**	
**Age**		**43 (12)**		**43 (12)**
Education level				
More than 4 years of higher education	9%		37%	
Up to 4 years higher education	58%		32%	
High school^a^	14%		19%	
Not finished high school	20%		12%	
Education				
Nurse	40%		13%	
Physician	5%		28%	
Assistant nurse	2%		1%	
Administration and economy	2%		4%	
Other	51%		55%	
Income^b, c^				
Low (<200)	30%		21%	
Mid-low (200–300)	32%		21%	
Average (300–400)	24%		30%	
Mid-high (400–500)	7%		16%	
High (>500)	6%		12%	
Absence during a year				
No absence spells^d^	72%		84%	
One absence spell^d^	22%		13%	
Multiple absence spells^d^	7%		3%	
Absence from 2003 to 2008				
No absence spells^d^	42%		65%	
One absence spell^d^	20%		16%	
Multiple absence spells^d^	38%		18%	
Days lost per absent employees per year^e^		98 (82)		89 (81)

Note. The Table presents all employees in the sample, also those who had no absence spell.

^a^13/14 years of school including compulsory education of 10 years.

^b^The number is taken from the first month each individual was measured.

^c^Given in 1,000 NOK ≈ 130 EUR or 170 USD.

^d^Amount of absence spells longer than 16 days during a year.

^e^The average amount of days lost to sickness absence for an employee who is absent during a year.

### Measurement

Data were drawn from two sources: (1) hospital data were provided by the research institute, SINTEF [40-45]; (2) individual level data was provided by Statistics Norway’s register-based longitudinal “Events Database”. Using Registry-Based Employee Statistics from Statistics Norway, data on individual employees were merged with hospital-level data. Because of the sensitive nature of the data, the Regional Committee for Medical and Health Research Ethics (REC) approved the study before any data were made available to the research project team members.

#### Mergers

**Figure 1:**
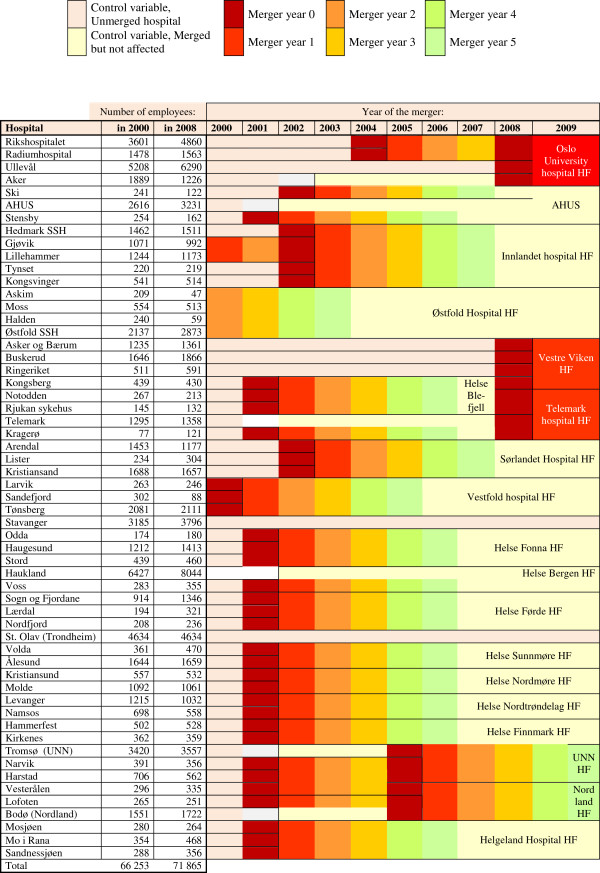
Hospitals included in the analysis.

Data on mergers are openly available and are presented in Figure 1. Mergers were defined by the year that the merged hospitals started to report data as a single hospital to the National Patient Register. For the analysis, six dummy variables were created to measure time since merger (year 0 to year 5). Year 0 was the year before the hospitals reported as a single unit; year 1 was the first year the hospitals reported as a unit; year 2 was the second year, and so on. The registration data reflected whether the hospitals were officially merged on January 1 of the given year. For several of the hospitals, the official merger date fell in year 0. Years within the study period (from 2000 to 2008), prior to year 0 and after year 5, were defined as non-merger years and were used as the reference group.

All mergers implied engagement of a common chief executive. However, the mergers differed, particularly regarding the extent of centralization of acute services, and the amount of change activities. Further, several hospitals were also involved in more than one merger during the study period. Figure 1 shows how the mergers were coded depending on when the units registered as a single unit. In some cases, the merger was between a small and large hospital. In those cases where a hospital was five or more times larger than the other hospital, we found that the merger was unlikely to affect the larger hospital. However, we expected to find an effect on the smaller hospital, so we defined it as a merger for the small hospital only. Figure 1 also shows the number of employees before and after the mergers. It is rare to observe a reduction in employee numbers; however, in some cases the smallest hospitals experienced a transfer of employees to larger hospitals. This was observed in the case of Aker, which merged with Oslo University Hospitals; Aker was gradually closed down as 2013 approached.

#### Sickness absence

Sickness absence was defined as a dichotomous variable measuring whether a person had been absent for more than 16 days, at least once during a year. Long-term sickness absence requires physician certification; this has been shown to be a better measure of ill health, rather than shorter spells [46]. Thus, our preferred measure of sickness absence was less prone to bias caused by non-health-related absence than short-term sickness absence. In addition, because sickness absence pay is covered by the state from day 17 of absence, the registry for absences of this length was complete, with no missing data.

#### Control variables

The year-specific variable was considered to be a linear variable for time, to control for the general increase in long-term sickness absence over the study period. This variable also controlled for any linear increase in absence as the participants grew older.

Reforms during 2004 to the Norwegian sick pay scheme placed stricter requirements on patients, doctors, and employers. The reform included six elements: eg the doctor’s first choice should be graded sick leave; the doctor should initiate work-related activity by 8 weeks (and if not, a written medical justification should be given to the authorities); and doctors who fail to comply with the new rules can be sanctioned by being stripped of the right to issue sick leave certificates. Immediately after the implementation of these reforms, sickness absence rates dropped by 23 per cent [46]. A legislation-specific variable was a dummy variable for every year after 2003, and was included to control for this legislative change.

### Statistical analysis and specification

Variation in long-term sickness absence was explained through a fixed effects multivariate regression analysis, with years since merger as the independent variable. An important advantage of this method is that it allowed us to use the longitudinal data to isolate within-employee effects, i.e. each employee’s odds of entering long-term sickness absence during merger years was compared only with their own odds of entering long-term absence in non-merger years. This method was useful for controlling for all of the differences that stayed constant over time between individuals and hospitals; it inherently controls for known differences (such as gender and education), but also unknown differences between employees that could have a larger effect. Examples of scenarios that could lead to inflated or underestimated effects when comparing between-individuals include: 1) some hospitals experience a greater number of mergers and have more unhealthy employees; 2) unhealthy employees leave more frequently at the beginning of a merger, and therefore experience fewer merger years.

A drawback of this fixed effects method is that because we focused solely on changes within employees, we lost information (i.e. all between-individual differences). The loss of this information may have caused increased confidence intervals and standard errors. We believe that this loss of information is justified because the lost data was more likely to have been confounded by between-individual differences. Another drawback of the statistical analyses is that while the method controlled for between-individual differences, the method did not provide estimates of their relative importance. To analyze the effect of gender, education and so forth, other methods, such as random effects, may have been more suitable.

We used logistic fixed effects regression to analyze the odds of entering long-term sickness absence each year. We chose logistic regression, rather than Poisson regression (i.e. analyzing the incidence rate of absence spells) because most employees had only 0 or 1 long-term absence each year, and the method was not influenced by high zero-inflation and over-dispersion in the sickness absence data.

## Results

The results from the analysis are presented in Tables 2 and 3. The results from the fixed effects analysis of the hospital merger effect shows an increase in the odds of long-term absence in year 0; this was the final period when the hospitals were still separate entities, but also represents the first period of the merger. The odds returned to normal after the first year and increased in years 2, 3 and 4.

**Table 2:** Odds ratios of long-term sickness absence before and after hospital mergers

	**OR**	**S.E.**	
Non-merger years during 2000-2008	1	-	
Year 0	1.05	0.02	**
Year 1	1.02	0.02	
Year 2	1.04	0.02	*
Year 3	1.07	0.02	***
Year 4	1.09	0.02	***
Year 5	1.04	0.02	
Year-specific variable		Yes	
Legislation-specific variable		Yes	

Note. The Table presents the employees’ odds ratio of entering long-term sickness absence each year from the year prior to reporting as one merged hospital to 5 years after the merger. The analyses were done with fixed effects so that each employee’s odds during the merger years were compared with the same employee’s odds the years prior to and after the merger years within the study period, 2000–2008 (baseline).

N employees: 47,485; N employee-years: 292,990.

*p < 0.05 **p < 0.01 ***p < 0.001.

**Table 3:** Odds ratios of long-term sickness absence before and after hospital mergers

		**Female**			**Male**	
	**OR**	**S.E.**		**OR**	**S.E.**	
Non-merger years during 2000–2008	1			1		
Year 0	1,04	0,02	*	1,05	0,05	
Year 1	1,03	0,02		0,93	0,05	
Year 2	1,05	0,02	*	0,98	0,05	
Year 3	1,08	0,02	***	0,96	0,05	
Year 4	1,08	0,02	**	1,17	0,07	*
Year 5	1,04	0,02		1,05	0,07	
Year-specific variable	Yes			Yes		
Legislation-specific variable	Yes			Yes		

*Note. The Table presents the employees’ odds ratio of entering long-term sickness absence each year from the year prior to reporting as one merged hospital to 5 years after the merger. The analyses were done with fixed effects so that each employee’s odds during the merger years were compared with the same employee’s odds the years prior to and after the merger years within the study period, 2000*–*2008 (baseline).*

N employees female: 40 973; N employee-years: 253 324.

N employees male: 6 505; N employee-years: 39 605.

*p < 0.05 **p < 0.01 ***p < 0.001.

For the analyses separated by gender, the results are replicated for female employees, with significantly higher long-term sickness absence in year 0, 2, 3 and 4. For men, the odds of entering long-term sickness absence were only significantly higher in year 4.

The odds ratio for women can be interpreted as between a 4.3% increase in odds in year 0, and an 8.2% increase in year 4. How much these impact the absolute figures for hospitals depends on the existing sickness absence levels at the hospital. However, for a female employee with an average likelihood of entering long-term sickness absence of 28.2% in non-merger year, the likelihood would increase by 1.6 percentage points to 29.8%. This equates to approximately 16 more employees entering long-term sickness absence per 1000 female employees in year 4 alone; once absent from work, on average, each women will be absent more than 3 months that year.

## Discussion

Our analysis shows that hospital mergers have a significant effect on long-term sickness absence, supporting a negative health effect from mergers on employees. The results indicate that mergers have a particularly significant effect on sickness absences during the initial phase of the merger. However, the effect was not significant after a year; the workers seemed to adapt to their new work situations very quickly. Conversely, 2 to 4 years after mergers, the odds of long-term sickness absence were significantly higher than normal. This effect may be explained by the internal changes that take place after mergers. Other studies have shown that merging hospitals have a higher degree of internal organizational change compared with stable hospitals [47], and that the main intention of mergers is to trigger such change [48,49]. We assume that any internal changes should be implemented 2 to 3 years into the merger, and this explains the significant effect of mergers in later years. It is likely that we are observing an indirect effect of mergers.

It has been reported that sick leave is higher in larger organizations. Voss, Floderus, and Diderichsen [50] found that employees in work places with more than 50 employees had a moderately higher risk of sick leave than employees in smaller work places. One could argue that this may be the reason why sick leave rates remained high 2 to 5 years after the merger in our study. However, the fact that most of the work places in our study employed more than 50 people before the merger took place weakens this interpretation. When the analyses were divided by gender, the results showed that hospital mergers have a significant effect on female employee long-term sickness absence for year 0, and years 2 to 4 after the merger; this supports a negative health effect of mergers on female employees. The analysis for male employees showed significantly higher sickness absence in year 4 only, which partially supports a negative effect on men. It is plausible that females may react more adversely to mergers than men. In Norway, females have higher rates of self-certified and medically certified absence, even after adjusting for pregnancy-related absenteeism [10,11]. A literature review of the relationship between gender and sickness absence, reveals that the psychosocial work environment might influence sickness absence of women differently than men; women might react differently to stressors, use different resources, and use absence as a coping mechanism to a greater extent [12]. However, with clearly fewer men, and less variation in the dependent variable (due to less absence among men), we cannot exclude a similar effect on men using the current findings.

Our results are consistent with studies that have shown negative health consequences from the announcement of, and during, a merger [19,28,30]. Our results are not congruent with the findings of Westerlund, Ferrie, Hagberg, Jeding, Oxenstierna and Theorell [31] who found no significant effect of mergers on long-term sickness absence after merger. Compared with our study, these authors [31] focused on longer absence spells (90 days or more, versus 17 days or greater in the present study), possibly making the current study more sensitive to more moderate effects. Perhaps more importantly, they focused on the effects after merger (from 2 months to 9 years later). In the current study we focused on sickness absence prior to and during merger (up to 5 years after implementation). Ours results indicate that the effects of merger are not significant 5 years after merger. Therefore, the timing of the measure might be crucial in relation to the ability to detect effects. Future studies should attempt to include even longer time spans than used in the present study, to investigate this assumption.

Our results indicate that mergers and the quest for higher productivity may come at a price, i.e. higher levels of long-term sickness absence. Mergers are initiated by regional health authorities to improve the production, distribution and organization of healthcare services. If the effects of mergers and subsequent internal changes include increased long-term sickness absence, then mergers should be seen as counterproductive. The effect of such mergers, which entails between a 4% and 8% increase in the odds of entering long-term sickness absence, could be viewed as modest. However, the seriousness of the output variable implies that this effect should be interpreted as being meaningful and relevant. For hospitals and the state, long-term sickness absence represents a large monetary loss, and a loss of workforce capacity. Employees are compensated with full pay for up to a year [51]. The additional costs related to replacing the employee, and from productivity losses, is estimated to be 40% for a registered hospital nurse [52]. The long-term sickness absence of an individual employee is generally a symptom of an increased risk of serious health impairments [7]. Additionally, long-term absence from work may have negative consequences, including: alienation, feelings of guilt, reduced well-being and enthusiasm for work, hindered career and salary development, and increased risk of exiting the workforce [53-56].

Mergers can be very disruptive for the work environment because they are often combined with heavy layoffs. However, the mergers we analyzed did not result in layoffs. In fact, the total number of hospital employees grew during the study period, even in most of the merged hospitals (see Figure 1). For this reason, our findings are especially interesting. However, we still do not know exactly what merger characteristics are stressful and exhausting and lead to higher sickness absence. We believe the answer might be found by looking closely at the factors related to changes in the psychosocial working conditions and perceived threats to current job tasks.

### Limitations

In the present study, we followed 57 hospitals over 8 years through more than 20 mergers. Although this was a unique dataset, it limited our ability to gain detailed knowledge about specific change processes. We do not know precisely when the mergers occurred. We do not know the consequences that the different events had on working conditions at the different hospitals, or how they were experienced by employees. Mergers have different approaches and impacts; the findings described here are an average. Although we did not have the official merger dates, the fact that people generally reacted to such changes prior to implementation makes the official date less important [15,57].

The reference group (the non-merging hospitals) consisted of years before the merger and six years or later after the merger. It could be argued that the effect of the merger could still be present after year five. However, it was important to include some time after the merger in the reference group, since we was interested in the effect of the change process due to the merger. In a longer time span there would be a risk that the effect is due to changes other than the merger. Several of the hospitals was already engaged in a second merger before year six after the first merger (Figure 1). We assumed that after five years the effect would no longer be due to the spesific mergers and therefore was included in the refence group of non-merging hospitals (unless the hospitals was engaged in a second merger).

We considered long-term sickness absence as a measure of ill health. Although there are several advantages with this measure, some potential limitations must be considered. The first potential limitation is sickness presence, i.e. employees attending work while ill [58]. During organizational change, increased job uncertainty [59] can be a factor that increases attendance pressure. Job uncertainty compels employees to attend work while ill, even if they qualify for medically-certified absence [60-62]. Job uncertainty challenges can, therefore, constrain absence levels during organizational change [61,63] and lead to an underestimation of this effect. However, layoffs are rare in Norwegian hospitals, even with instances of redundancy [64]; the mergers in our study were seldom accompanied by major downsizing. While it is possible that fears of being moved, or of a temporary contract not being renewed, may have had a similar effect on employees, the confounding due to job insecurity-related sickness presence is probably relatively small.

Another potential limitation of focusing on long-term sickness absence is the loss of information relating to shorter absence spells. Although shorter spells of absence are more likely to be affected by factors other than health [9], we might have simultaneously lost information about shorter periods of illness that were closely related to merger strain and fatigue. While focusing on long-term absence may have reduced potential confounding variables, such as reduced satisfaction, we could have also underestimated the total effect of the mergers.

The lack of significant results among men might in part be due to an occupational effect. Even though the majority of the physicians are woman, a higher proportion of the men in the sample are physicians, 28% of men versus 5% of women (Table 1). Very few men are nurses and nurses represent the majority of the employees (34.5%). Similarly a higher proportion of men have more than 4 years of higher education, 37% of men versus 9% of women. If physicians, and other professions with high education, are less likely to respond to a merger by increased long-term absence, it would reduce the possibility of a significant effect in the male sample. It is however worth nothing that approximately the same proportion of employees did not have any higher education in the male and female sample, 31% of men and 34% of women. Additionally, while the sample was generally heterogeneous, the majority of participants were female. Further, more men than women had no reported long-term absence spells during the study period, and therefore did not provide information for the analyses. It is probable that we did not have the necessary statistical power to estimate the effect of mergers on the male cohort. Further studies are needed to examine the effect of mergers on men or the occupational effect.

## Conclusions

The results of our study showed an increased long-term sickness absence, prior to and after hospital mergers, particularly in female employees. The effect might be viewed as moderate. However, because of the seriousness of the dependent variable, it is significant enough that it should be considered when health managers plan to merge hospitals. This study illustrates the importance of analyzing the effects of mergers over several years. Other studies have also shown how mergers affect hospitals over a longer time span [4,22,48,65].

Mergers have an inconclusive effect on cost-efficiency [2,65,66]. This study shows that mergers can also affect long-term sickness absence among hospital employees. It also indicates that there is a need for more detailed analyses of merger processes and of the changes and conditions that may occur as a result of such mergers.

## Competing interests

The authors declare that they have no competing interests.

## Authors’ contributions

LEK - Conceived of the study, acquisition of data, analysis and interpretation of data, drafted the manuscript. VHB - Participated in the design of the study, performed the statistical analysis, interpretation of data and critical revision of paper. ED - Analysis and interpretation of data and critical revision of paper. TL - Analysis and interpretation of data, critical revision of paper, responsible for preparing the Statistics Norway’s register-based longitudinal “Events Database” for analysis. All authors read and approved the final manuscript.

## Pre-publication history

The pre-publication history for this paper can be accessed here:

http://www.biomedcentral.com/1472-6963/14/50/prepub
